# Editorial: Physical-chemical interactions and composition-structure-property modifications during processing: Food quality, nutrition, and health

**DOI:** 10.3389/fnut.2022.1044382

**Published:** 2022-10-18

**Authors:** Qiang Xia, Brain D. Green, Zhonghua Liu

**Affiliations:** ^1^Key Laboratory of Animal Protein Food Processing Technology of Zhejiang Province, College of Food and Pharmaceutical Science, Ningbo University, Ningbo, China; ^2^Institute for Global Food Security, Queen's University Belfast, Belfast, United Kingdom; ^3^Key Laboratory of Tea Science of Ministry of Education, Hunan Agricultural University, Changsha, China

**Keywords:** composition-structure-property, thermal treatments, nonthermal processing, process development and optimization, physicochemical interactions

Recent years have witnessed the role of industrial food processing in driving the creation and transformation of a modernized and facilitated lifestyle by providing efficient food supply chains and sustainable diets (1–3), particularly for those effective, eco-friendly and energy-saving pretreatment or manufacturing technologies. Different food unit operations aim at assuring physicochemical stability and microbiological and nutrition safety, and simultaneously obtaining desired modifications in the composition and structure of food matrices, accompanied by positive health implications (4). Therefore, the correlation between extrinsic processing factors and composition-structure-properties response has been one of major focuses of food science research, largely relying on food matrices and the applied technique types (5, 6). In this regard, thermal conditions receive the most attention considering their generalization and high applicability. For current food industries, multiple processing techniques, process optimization strategies, and the exploitation of new food sources have been largely developed. Resultantly, the presence of new-type physicochemical interaction behavior and phenomenon among minor/major components and composition-structure-property relation modifications exerted by emerging processing methods or patterns or in new combination patterns require more elaborate characterization, analysis, and summarization over the response of quality, nutritional and health properties of final products, in order to achieve the tailored production of nutritional and health foods (7, 8). Particularly, healthier food can be obtained *via* suitable food re-formulation and microstructure designing (9), relying on the accumulation of knowledge about the correlation between food structure, the gastrointestinal fate of nutrients and satiety response.

The current Research Topic aims at highlighting the progress and roles of processes-induced physicochemical modifications and interaction behavior of different intrinsic food components, particularly at molecular levels, in regulating the changes of quality, storability, nutrition and health characteristics of food products. In this collection, a total of 13 papers have been published, related to the development of analysis, evaluation and characterization techniques for the complicated network of chemical and compositional differences, machine imaging, introduction and summarization of the newly developed processing techniques and the effects, as well as the processing and nutrition attributes of food materials with new origins (Jia, Chu et al., Hong et al., Ma et al.).

Novel characterization techniques and methodologies applied to monitor compositional variations or differences during processing are expected in this Research Topic. Li et al. summarized advanced lipidomics applied to muscle origin differentiation and meat processing, with the aspects of quality traceability, processing requirement, and health concerns involved. Cai et al. reported a two-tube hexaplex polymerase chain reaction technique used for molecular authentication which may occur in commercial meat processing. Another mini-review performed by Xiao, Wang et al. reported the application of a machine vision system for food quality monitoring during processing. Effects of novel processing techniques and varied combination patterns on the chemical modifications during processing are also reported in this collection, as observed in the cases of plasma-activated water (Yan et al.), hydrodynamic cavitation (Sun et al.), and photolysis (Xiao, Xuan et al.). These cases suggested that the major food physicochemical attributes during processing and transportation can be modified significantly, depending on many process parameters, as exemplified by the correlation between food safety properties and rotary motion conditions (e.g., vibration, noise, and temperature rise) of food transport pumps (Jia, Li, et al.).

In conclusion, it can be inferred from these papers of this collection that scientific and technical challenges during food processing have been overcome to a large degree, with both novel processing techniques and detection/characterization strategies developed. However, there are some research aspects that should be highlighted in future related work, as exemplified by the following items: (i) metabolic and omic characterization of bio-processing foods; (ii) interaction patterns between food components as affected by processing parameters, as well as its effects on nutritional properties (e.g., digestibility, bioaccessibility, bioavailability, etc.); (iii) comparison of chemical modifications of food components (e.g., protein, lipid, carbohydrates, phytochemicals, etc.) between traditional thermal processing and emerging nonthermal processing processes or the combined patterns.

## Author contributions

QX: drafted the manuscript. BG and ZL: conceptualized, reviewed, and edited the manuscript. All authors contributed to the article and approved the submitted version.

## Funding

This work is financially supported by the National Natural Science Foundation of China (32101862) and Natural Science Foundation of Zhejiang Province (LQ20C200007).

## Conflict of interest

The authors declare that the research was conducted in the absence of any commercial or financial relationships that could be construed as a potential conflict of interest.

## Publisher's note

All claims expressed in this article are solely those of the authors and do not necessarily represent those of their affiliated organizations, or those of the publisher, the editors and the reviewers. Any product that may be evaluated in this article, or claim that may be made by its manufacturer, is not guaranteed or endorsed by the publisher.
